# Computational Analysis of Nanoparticle Shapes on Hybrid Nanofluid Flow Due to Flat Horizontal Plate via Solar Collector

**DOI:** 10.3390/nano12040663

**Published:** 2022-02-16

**Authors:** Muhammad Imran, Sumeira Yasmin, Hassan Waqas, Shan Ali Khan, Taseer Muhammad, Nawa Alshammari, Nawaf N. Hamadneh, Ilyas Khan

**Affiliations:** 1 Department of Mathematics, Government College University, Faisalabad 38000, Pakistan; drmimranchaudhary@gmail.com (M.I.); yasmeensumaira.1122@gmail.com (S.Y.); shazukhan1214@gmail.com (S.A.K.); 2 Department of Mathematics, College of Sciences, King Khalid University, Abha 61413, Saudi Arabia; tasgher@kku.edu.sa; 3 Department of Basic Sciences, College of Science and Theoretical Studies, Saudi Electronic University, Riyadh 11673, Saudi Arabia; n.alshammari@seu.edu.sa; 4 Department of Mathematics, College of Science Al-Zulfi, Majmaah University, Al-Majmaah 11952, Saudi Arabia; i.said@mu.edu.sa

**Keywords:** second grade hybrid nanofluid, entropy generation, thermal radiation, solar collector, nanoparticle shapes, bvp4c, MATLAB

## Abstract

The present work discusses the 2D unsteady flow of second grade hybrid nanofluid in terms of heat transfer and MHD effects over a stretchable moving flat horizontal porous plate. The entropy of system is taken into account. The magnetic field and the Joule heating effects are also considered. Tiny-sized nanoparticles of silicon carbide and titanium oxide dispersed in a base fluid, kerosene oil. Furthermore, the shape factors of tiny-sized particles (sphere, bricks, tetrahedron, and platelets) are explored and discussed in detail. The mathematical representation in expressions of PDEs is built by considering the heat transfer mechanism owing to the effects of Joule heating and viscous dissipation. The present set of PDEs (partial differential equations) are converted into ODEs (ordinary differential equations) by introducing suitable transformations, which are then resolved with the bvp4c (shooting) scheme in MATLAB. Graphical expressions and numerical data are obtained to scrutinize the variations of momentum and temperature fields versus different physical constraints.

## 1. Introduction

Nanoparticles are commonly employed in food, medication, nuclear power stations, agriculture, and other applications. Such fluids are created from the continuous dispersion of nano-sized particles in base fluids including water, ethylene glycol, lubricating oils, blood, or other fluids, and are termed as nanofluids. Hybrid nanofluids are formed by the dispersion of two or more components in the given base fluid. Such fluids have numerous applications in medicine research and technology. The majority of medications are manufactured as hybrid nanofluids, and blood is utilized as a research base fluid to assess the chemical interactions of the compounds in the blood. In addition, the hybrid-type nanofluids are employed to improve the thermal efficiencies of base fluids. Correct viscosities as well as temperatures are necessary to maintain the consistency of blood transfer for improved blood circulation. To improve the thermal properties of these fluids, nano-scale particles are dispersed in the base liquid, which improves the thermal attributes. Choi [1] has developed a comprehensive strategy for increasing the heat transfer rate of these fluids. Nanofluids are fluids with increased thermophysical characteristics. Choi’s pioneering study was motivated by the observation that base fluids with lower thermal conductivity are inefficient for industrial heat transfer applications. Chemicals, metals such as (Cu, Al and Ag), metallic compounds (SiC), metallic oxides (silica oxides, alumina oxides, and zirconia oxides), and nitrides are among the nanometer-sized particles that contribute to nanofluids. Nanofluids are classified into four types based on their natural environment and quantity: (i) nanofluids for pollution purifying (agricultural), (ii) nanofluids for heat transfer, (iii) nanofluids for drug delivery (healthcare fields), and (iv) pharmaceutical nanofluids with multiple effects in fields such as oncology, microbiology, and cardiology. Rasool et al. [2] explored the Marangoni convection in Casson-based nanofluid flow when impacted by the existence of Lorentz forces. Mahanthesh et al. [3] discovered the consequences of the quadratic thermal radiated effect and the quadratic Boussinesq assumptions on the heat transmission of 36 nano-sized nanoparticles across a vertical surface. Ramzan et al. [4] evaluated the magneto Casson-type nanofluid with a changing heat source/sink as well as modified Fourier’s and Fick’s laws (FFLs) across a stretched cylinder. Eid et al. [5] identified the hydrothermal differences of viscous and elastic nanofluid flows in a porosity medium through a stretched surface. The behavior of Fourier’s and Fick’s laws on nanofluid flow was scrutinized by Gowda et al. [6]. Alsabery et al. [7] disclosed the convection phenomenon in nanofluid flow. Alsabery et al. [8] investigated the forced convection heat transformer through horizontal channels. Nanofluid investigation has been further addressed in different recent works [9,10,11,12].

**Figure 1:**
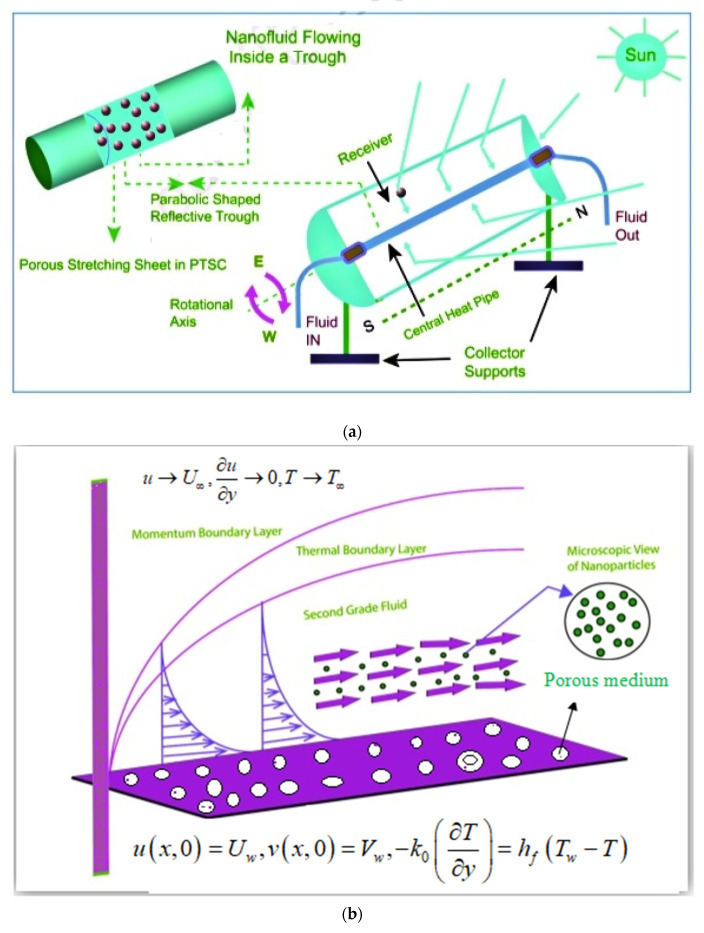

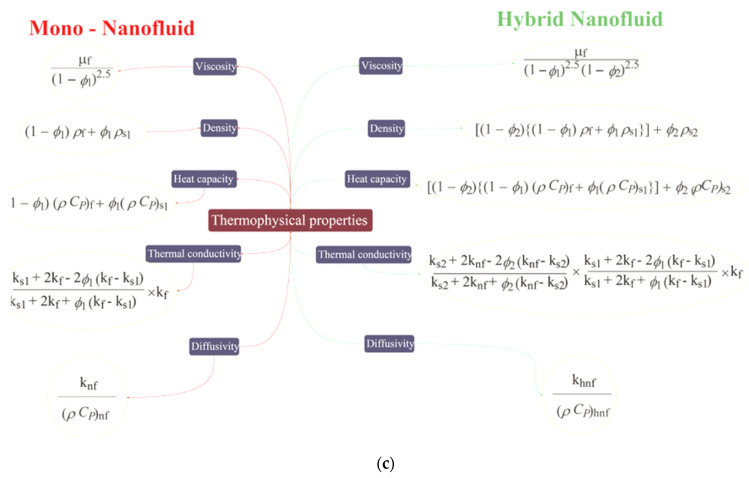
(**a**) solar collector system with representation, (**b**) schematic of flow problem, and (**c**) thermophysical properties of nanofluid and hybrid nanofluid.

A solar energy collector (SEC) (see Figure 1a) is defined as a heat transfer mechanism that absorbs and transforms solar radiation into heat, which is then transported to a fluid (commonly $H_{2}O$, air, or lubricant) that moves via an SC (solar collector). Electric energy for manufacturing purposes necessitates the use of large capacity solar collectors. However, the mechanism is inefficient due to poor heat transport. As a consequence, a great deal of study has been conducted in fields including heat pumps, hot water services, cooling, and manufacturing processes. Solar energy is a natural and abundant source of energy. Due to the tremendous utility of solar energy, it is employed in implementations such as solar collectors. The implementations of nanofluids to enhance the efficiency of solar thermal collectors over standard Newtonian fluids are well established within research. Hayat et al. [13] reported the three-dimensional second grade boundary layer flow of nanofluids through a stretched plate including thermal radiation with a heat source/sink effect. Waqas et al. [14] considered the bio-convective thermal radiative impact in Darcy–Forchheimer nanofluid flow with Wu’s slip through an expanding cylinder/plate. Wakif et al. [15] scrutinized the thermal radiation impact on aluminum–copper oxide hybrid-based nanofluids. Hussain et al. [16] assessed the heat transformer characteristics of MHD (magnetohydrodynamic) with hybrid-based nanofluid flow in the existence of thermal radiation effects. Kerschbaumer et al. [17] established that radiation refrigeration is a relevant issue for applications in thermal management of buildings as well as energy conservation. Alsabery et al. [18] discussed the dual phase nanofluid in a 3D solar collector. Han and Chen [19] discussed the micro-nanofluidic preconcentrator through a microchannel. Han and Chen [20] discussed the implementations of ion transport in micro-nanofluidic mechanisms. Waqas et al. [21] investigated the bioconvective flow of cross nanofluid in the presence of activation energy. Han and Chen [22] discussed viscous nanofluids in microchannels. The graphical representation is elucidated in Figure 1a.

In recent decades, researchers and engineers have placed considerable attention on entropy generation. Due to their significance, the characteristics and importance of entropy in various industry applications such as electrical heaters, refrigerators, combustion generators, have been investigated in the context of most aspects of heat phenomena. Entropy generation minimization is a method for modeling and optimizing equipment that have (thermodynamic) inefficiencies because of several properties, such as fluid flow viscous dissipation. As a result, for more advantageous execution of any structure, the conditions that would be effective in entropy generation depreciation should be identified. Entropy generation investigation is useful in analyzing the reliability of electrical or mechanical devices. Fluid dynamics include a combination of fundamental irreversible behavior, fluid viscosity with mechanism, and Joule heating, etc. Eid et al. [23] explored the 2D cross nanofluid flow through a linearly stretchy surface with a magnetic field in the Darcy–Forchheimer permeable regime. Eid et al. [24] examined the carbon nanotubes *CNT’s* suspending magnetohydrodynamics flow of micropolar dusty nanoparticles impinging on a porous extended surface inserted in a porous regime. Turkyilmazoglu et al. [25] characterized the velocity slip effect and entropy generation in thermal radiative transportation via a metal porous channel. Shehzad et al. [26] investigated how the heat flow in microchannels involving entropy generation can be useful in many implementations including micro-aircrafts, mechanical–electromechanical solutions, electrical device refrigeration, and micro-heat transfer mechanisms. Hayat et al. [27] analyzed the Carreau nanofluid flow with entropy generation.

From the above discussed literature and other similar research, the authors of the current research observed that few studies are available of second grade hybrid nanofluid flow (HNF) through a stretched surface. The current work aims to clarify the impacts of nanoparticle shape factors on second grade hybrid nanofluid over a flat horizontal porous surface with entropy generation. The significance of thermal radiation and viscous dissipation on heat equation is scrutinized. The velocity slip and convective heat transfer are also analyzed. The nanoparticles silicon carbide SiC and titanium oxide $TiO_{2}$ are involved in kerosene oil-based fluids to improve the heat transfer. In the current research, the bvp4c tool in MATLAB is used to find the solution. This crucial research may help to enhance industrial production, especially in the solar energy collector sectors. This research is more applicable in the field of heat transfer, and current outcomes may be more effective in nanotechnology and biomedical fields such as drug delivery and cancer treatment. Therefore, such computational analysis is attractive to researchers.

## 2. Mathematical and Physical Descriptions

Here, we consider the steady, two-dimensional second grade laminar flow with a hybrid-type nanofluid model across a stretched porous surface in the existence of different nanoparticle shapes. Furthermore, the effects of radiative heat flux in the occurrence of a viscous dissipation effect and Joule heating are addressed. Moreover, the magnetic field is examined. The entropy of system is also involved in the current study. The present model was sketched with mathematical characteristics which indicate the fluid velocity, held smooth, and the stretching surface under the irregular extending rate:

(1) $$U_{w}(x,t)=bx,$$

where b represents the preliminary extendable rate of a stretched porous surface. The isolated temperature of a flat surface is symbolized by $T_{w}(x,t)=T_{\infty }+b^{*}x$ and it is considered that its suitability is constant in supposition at x=0, $\left(b^{*},T_{w}\&T_{\infty }\right)$, describing the thermal variation rate as well as fluid temperature of wall and ambient temperature respectively.

Additionally, the stress-tensor of Williamson nanofluids is expressed as:

(2) $$S^{*}=\mu A_{\zeta 1}+\alpha_{1}A_{\zeta 2}+\alpha_{1}A_{\zeta 1}^{2}-pI,$$

where the additional stress tensor in a second grade is symbolized by $A_{ij}$ and, mathematically, the form is expressed as:

(3) $$\begin{array}{l} A_{\zeta 1}=\left(grad\;V\right)+{\left(grad\;V\right)}^{T},\\ A_{\zeta 2}=\frac{dA_{\zeta 1}}{dt}+A_{\zeta 1}\left(grad\;V\right)+A_{\zeta 1}{\left(grad\;V\right)}^{T}, \end{array}$$

where $\left(\alpha_{1}\&\alpha_{2}\right)$ represents the material variables. μ is denoted by the fluid dynamic viscosity, p represents the pressure,I explains the identity tensor, $\left(A_{\zeta 1}\&A_{\zeta 2}\right)$ both demonstrate the Rivlin–Erickson tensors, $\frac{d}{dt}$ expresses the time-dependent derivative, and the fluid velocity is signified by V. We prove the Clausius–Duhem inequality. Furthermore, we determine that the Helmholtz unlimited temperature is the minutest in equipoise for the liquid flow closed by at rest when:

$$\mu \geq 0,\alpha_{1}\geq 0,\alpha_{1}+\alpha_{2}=0$$

If $\alpha_{1}+\alpha_{2}=0,$ then the second grade nanofluid expression is diminishable from the viscous fluid. Figure 1b shows a graphical demonstration of the current theoretical observation.

### 2.1. Governing Equations

The flow problem which involves the set of governing equations, such as velocity and temperature, is expressed as [28,29]:

(4) $$\frac{\partial u}{\partial x}+\frac{\partial v}{\partial y}=0,$$

(5) $$\begin{array}{l} u\frac{\partial u}{\partial x}+v\frac{\partial u}{\partial y}=U_{\infty }\frac{dU_{\infty }}{dy}+\frac{\alpha_{1}}{\rho_{hnf}}\left[\frac{\partial u}{\partial x}\left(\frac{\partial^{2}u}{\partial y^{2}}\right)+u\left(\frac{\partial^{3}u}{\partial x\partial y^{2}}\right)+\frac{\partial u}{\partial y}\left(\frac{\partial^{2}v}{\partial y^{2}}\right)+v\left(\frac{\partial^{3}u}{\partial y^{3}}\right)\right]\\ +\frac{\mu_{hnf}}{\rho_{hnf}}\left(\frac{\partial^{2}u}{\partial y^{2}}\right)-\frac{\mu_{hnf}}{\rho_{hnf}k}u, \end{array}$$

(6) $$u\frac{\partial T}{\partial x}+v\frac{\partial T}{\partial y}=\frac{k_{hnf}}{{\left(\rho C_{p}\right)}_{hnf}}\left(\frac{\partial^{2}T}{\partial y^{2}}\right)-\frac{1}{{\left(\rho C_{p}\right)}_{hnf}}\left(\frac{\partial q_{r}}{\partial y}\right)+\frac{\mu_{hnf}}{{\left(\rho C_{p}\right)}_{hnf}}{\left(\frac{\partial u}{\partial y}\right)}^{2}+\frac{\sigma_{hnf}B^{2}\left(t\right)u^{2}}{{\left(\rho C_{p}\right)}_{hnf}}.$$

with boundary conditions:

(7) $$\begin{array}{l} u\left(x,0\right)=U_{w},v\left(x,0\right)=V_{w},T=T_{w},\\ u\to U_{\infty },\frac{\partial u}{\partial y}\to 0,T\to T_{\infty }\;\;\;as\;\;\;\;\;y\to \infty . \end{array}$$

In the above equations, the components of velocity are denoted by $\left(u\&v\right)$ along the $\left(x\&y\right)$ direction respectively, the dynamic viscosity of second grade hybrid nanofluid is $\mu_{hnf}$, $\left(\rho_{hnf}\right)$ represents the hybrid nanofluid density and $k_{hnf}$ shows the hybrid nanofluid thermal conductivity (see Table 1). Furthermore, $q_{r}$ considers the thermal radiative heat flux, ${\left(\rho C_{p}\right)}_{hnf}$ describes the specific heat capacitance of nanofluid, and the porous stretchable surface characterized by $V_{w}$.

Here, hnf explains the thermophysical aspect of the nanofluid, the solid particles are shown as s1,s2, the base fluid is represented as f, and the solid volume fraction nominated by $\phi_{1},\phi_{2}$ utilizing the nanoparticles. The thermal conductivity in case of shape factors is addressed as:

$$k_{hnf}/k_{gf}=\left[\left(\left(k_{s1}+\left(m-1\right)k_{gf}\right)-\left(m-1\right)\phi_{2}\left(k_{gf}-k_{s2}\right)\right)/\left(\left(k_{s2}+\left(m-1\right)k_{gf}\right)-\phi_{2}\left(k_{gf}-k_{s2}\right)\right)\right].$$

Here, $k_{gf}/k_{f}=\left[\left(\left(k_{s1}+\left(m-1\right)k_{f}\right)-\left(m-1\right)\phi_{1}\left(k_{f}-k_{s1}\right)\right)/\left(\left(k_{s1}+\left(m-1\right)k_{f}\right)-\phi_{1}\left(k_{f}-k_{s1}\right)\right)\right].$

### 2.2. Calculation of Rosseland Approximation

The Rosseland approximation by Brewster [30] can be expressed as:

(8) $$q_{r}=-\frac{4\sigma^{*}}{3k^{*}}\frac{\partial T^{4}}{\partial y},$$

where, $\sigma^{*}$ illustrates the Stefan–Boltzmann constant and $k^{*}$ stands for the mean absorption coefficient.

### 2.3. Similarity Transformations

The similarity transformation for PDEs which are converted into ODEs is addressed as:

(9) $$\zeta \left(x,y\right)=\sqrt{\frac{b}{\nu_{f}}}y,u=bxf^{\prime }\left(\zeta \right),v=\sqrt{\nu_{f}b}f\left(\zeta \right),\theta \left(\zeta \right)=\frac{T-T_{\infty }}{T_{w}-T_{\infty }}.$$

### 2.4. Resulting Equations

After applying the similarity transformations to the variables, the dimensional equations are reduced into dimensionless equation as follows:

(10) $$f^{\prime \prime \prime }+A^{2}+\Phi_{1}\left[\Phi_{2}\left(ff^{\prime \prime }-{f^{\prime }}^{2}\right)+\Gamma \left(2f^{\prime }f^{\prime \prime \prime }-{f^{\prime \prime }}^{2}-ff^{iv}\right)-\frac{Kf^{\prime }}{\Phi_{1}}\right]=0,$$

(11) $$\theta^{\prime \prime }\left(1+\frac{1}{\Phi_{5}}PrNr\right)+Pr\frac{\Phi_{3}}{\Phi_{5}}\left(f\theta^{\prime }-f^{\prime }\theta +\frac{Ec}{\Phi_{1}\Phi_{3}}{f^{\prime \prime }}^{2}+\frac{\Phi_{4}}{\Phi_{3}}M.Ec.{f^{\prime }}^{2}\right)=0,$$

with B.C.

(12) $$\begin{array}{l} f\left(0\right)=S,f^{\prime }\left(0\right)=1,\theta \left(0\right)=1,\\ f^{\prime }\left(\zeta \right)\to A,f^{\prime \prime }\left(\zeta \right)\to 0,\theta \left(\zeta \right)\to 0,\;\;as\;\;\zeta \to \infty . \end{array}$$

Here, $\phi_{i}{}^{\prime }s$ is 1≤i≤5 in the velocity and temperature equation of second grade hybrid nanofluid.

(13) $$\begin{array}{l} \Phi_{1}={\left(1-\phi_{1}\right)}^{2.5}{\left(1-\phi_{2}\right)}^{2.5},\Phi_{2}=\left(1-\phi_{2}\right)\left(\left(1-\phi_{1}\right)+\phi_{1}\frac{\rho_{s1}}{\rho_{f}}\right)+\phi_{2}\frac{\rho_{s2}}{\rho_{f}},\\ \Phi_{3}=\left(1-\phi_{2}\right)\left(\left(1-\phi_{1}\right)+\phi_{1}\frac{{\left(\rho C_{p}\right)}_{s1}}{{\left(\rho C_{p}\right)}_{f}}\right)+\phi_{2}\left(\frac{{\left(\rho C_{p}\right)}_{s2}}{{\left(pC_{p}\right)}_{f}}\right),\Phi_{4}=\frac{\sigma_{hnf}}{\sigma_{f}},\\ \Phi_{5}=\left(\frac{\left(k_{s2}+\left(m-1\right)k_{gf}-\left(m-1\right)\phi_{2}\left(k_{gf}-k_{s2}\right)\right)}{\left(k_{s2}+\left(m-1\right)k_{gf}+\phi_{2}\left(k_{gf}-k_{s2}\right)\right)}\right)\left[\frac{\left(\begin{array}{l} k_{s1}+\left(m-1\right)k_{f}\\ -\phi_{1}\left(k_{f}-k_{s1}\right) \end{array}\right)}{\left(\begin{array}{l} k_{p1}+\left(m-1\right)k_{f}\\ -\left(m-1\right)\phi_{1}\left(k_{f}-k_{s1}\right) \end{array}\right)}\right]. \end{array}$$

In Table 2, the geometrical form of nanoparticle shapes, their size, and sphericity are discussed in detail.

### 2.5. Non-Dimensional Parameters

The derivatives are signified with respect to ζ. The velocity ratio parameter, non-Newtonian second grade parameter, magnetic parameter, porous parameter, Prandtl number, thermal diffusivity, thermal radiation parameter, Eckert number, and suction/injection parameter are defined as follows:

(14) $$\left[\begin{array}{l} A=\frac{a}{b}\Gamma =\frac{\alpha_{1}b}{\mu_{f}},M=\frac{\sigma_{f}B_{0}^{2}}{c\rho_{f}},K=\frac{\nu_{f}}{bk},\;\mathrm{Pr}=\frac{\nu_{f}}{\alpha_{f}},\;\alpha_{f}=\frac{\kappa_{f}}{{\left(\rho C_{p}\right)}_{f}},\\ Nr=\frac{16\sigma^{*}T_{\infty }^{3}}{3k^{*}\nu_{f}{\left(\rho C_{p}\right)}_{f}},\;Ec=\frac{U_{w}^{2}}{\left(T_{w}-T_{\infty }\right){\left(C_{p}\right)}_{f}},S=-V_{w}\sqrt{\frac{1}{b\nu_{f}}}. \end{array}\right]$$

### 2.6. Physical Industrial Interest

In the present segment, the local skin friction coefficient and the heat transfer rate (local Nusselt number) of the flow problem are as follows:

(15) $$C_{f}=\frac{\tau_{w}}{\rho_{f}U_{w}^{2}},\;\;\;\;\;Nu_{x}=\frac{xq_{w}}{k_{f}\left(T_{w}-T_{\infty }\right)},$$

The complete shear stress with the heat flux $\left(\tau_{w}\&q_{w}\right)$ of wall is illustrated bellow:

(16) $$\left.\begin{array}{l} \tau_{w}={\left(\mu_{nf}\frac{\partial u}{\partial y}+\alpha_{1}\left(u\frac{\partial^{2}u}{\partial x\partial y}+2\frac{\partial u}{\partial y}\frac{\partial u}{\partial x}+v\frac{\partial^{2}u}{\partial y^{2}}\right)\right)}_{y=0},\\ q_{w}=-k_{hnf}\left(1+\frac{16}{3}\frac{\sigma^{*}T_{\infty }^{3}}{\kappa^{*}\nu_{f}{\left(\rho C_{p}\right)}_{f}}\right){\left(\frac{\partial T}{\partial y}\right)}_{y=0}, \end{array}\right\}$$

Here, the reduced forms of physical industrial material with drag force and heat transfer rate is shown as:

(17) CfRex12=f″0Φ1+Γ3f″0f′0−f‴0f0NuxRex−12=−knfkf1+Nrθ′0.

Here, local Nusselt numbers denoted by $Nu_{x}$ and $C_{f}$ represent the local skin friction coefficient. Finally, Rex=Uwxνf represents the local Reynolds number depending on the stretchable velocity $\left(U_{w}\left(x\right)\right)$.

### 2.7. Entropy of System

The dimensionless system of entropy is described as:

(18) $$N_{G}=Re\left(\Phi_{5}\left(1+Nr\right){\theta^{\prime }}^{2}+1/\Phi_{1}.Br./\Omega \left({f^{\prime \prime }}^{2}+K{f^{\prime }}^{2}\right)\right).$$

In which the Brinkman number is $Br=\varpi_{f}U_{w}^{2}/k_{f}\left(T_{w}-T_{\infty }\right)$, and $\Omega =T_{w}-T_{\infty }/T_{\infty }$ signifies the temperature gradient.

## 3. Numerical Algorithm (Shooting Scheme)

The flow system of ordinary differential Equations 10 and 11, under the specific boundary constraints 12, is solved numerically with the aid of the bvp4c method in MATLAB via the Lobattao-IIIa formula. For this phenomenon, firstly, the higher order ordinary differential equations are transmuted into first order ordinary differential equations (ODEs) with the help of innovative variables. Let

(19) $$\begin{array}{l} f=s_{1},\frac{df}{d\zeta }=s_{2},\frac{d^{2}f}{d\zeta^{2}}=s_{3},\frac{d^{3}f}{d\zeta^{3}}=s_{4},\frac{d^{4}f}{d\zeta^{4}}={s^{\prime }}_{4},\\ \theta =s_{5},\frac{d\theta }{d\zeta }=s_{6},\frac{d^{2}\theta }{d\zeta^{2}}={s^{\prime }}_{6}, \end{array}$$

(20) $${s^{\prime }}_{4}=\frac{-A^{2}+s_{4}+\Phi_{1}\left[\Phi_{2}\left(s_{1}s_{3}-s_{2}{}^{2}\right)+\Gamma \left(2s_{2}s_{4}-s_{3}{}^{2}\right)-\frac{Ks_{2}}{\Phi_{1}}\right]}{\Gamma \Phi_{1}s_{1}},$$

(21) $${s^{\prime }}_{6}=\frac{-Pr\frac{\Phi_{3}}{\Phi_{5}}\left(s_{1}s_{6}-s_{2}s_{5}+\frac{Ec}{\Phi_{1}\Phi_{3}}s_{3}{}^{2}+\frac{\Phi_{4}}{\Phi_{3}}M.Ec.s_{2}{}^{2}\right)}{\left(1+\frac{1}{\Phi_{5}}\mathrm{Pr}Nr\right)},$$

(22) $$\begin{array}{l} s_{1}\left(0\right)=S,s_{2}\left(0\right)=1,s_{5}\left(0\right)=1,\\ s_{2}\left(\zeta \right)\to A,s_{3}\left(\zeta \right)\to 0,s_{5}\left(\zeta \right)\to 0,\;\;as\;\;\zeta \to \infty . \end{array}$$

### Validation of Results

In this section, the validation of results is summarized. As shown in Table 3, we observed good agreement between published results and our current results.

## 4. Results and Discussion

**Figure 2:**
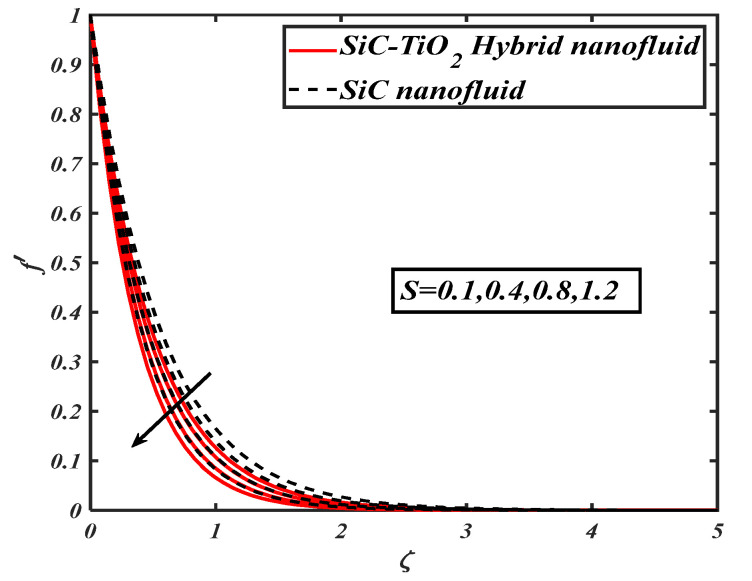
$f^{\prime }$ for varied S.

**Figure 3:**
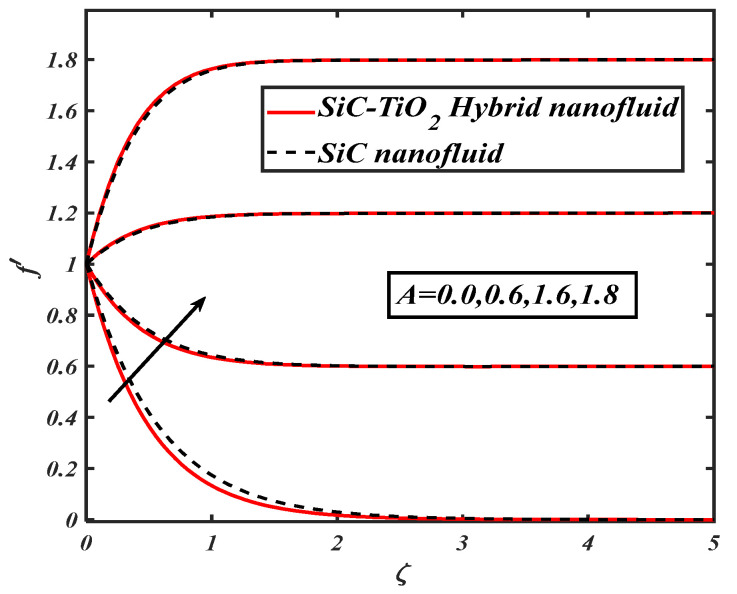
$f^{\prime }$ for varied A.

**Figure 4:**
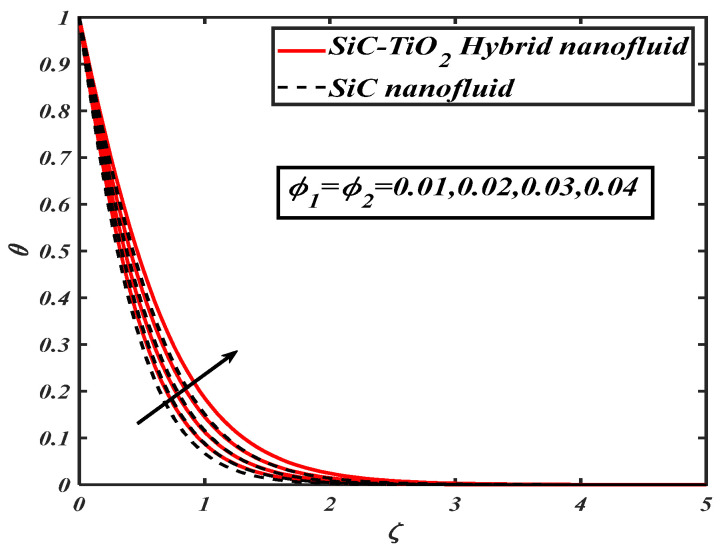
θ for varied $\phi ,\phi_{1}$.

**Figure 5:**
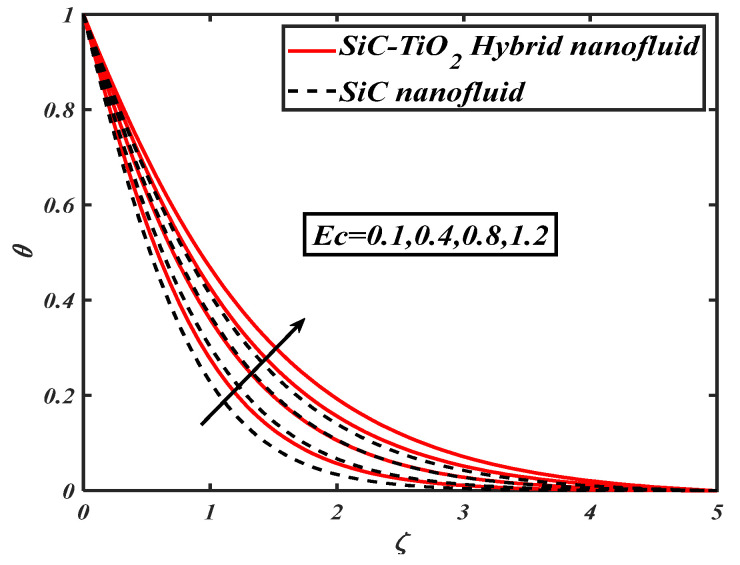
θ for varied Ec.

**Figure 6:**
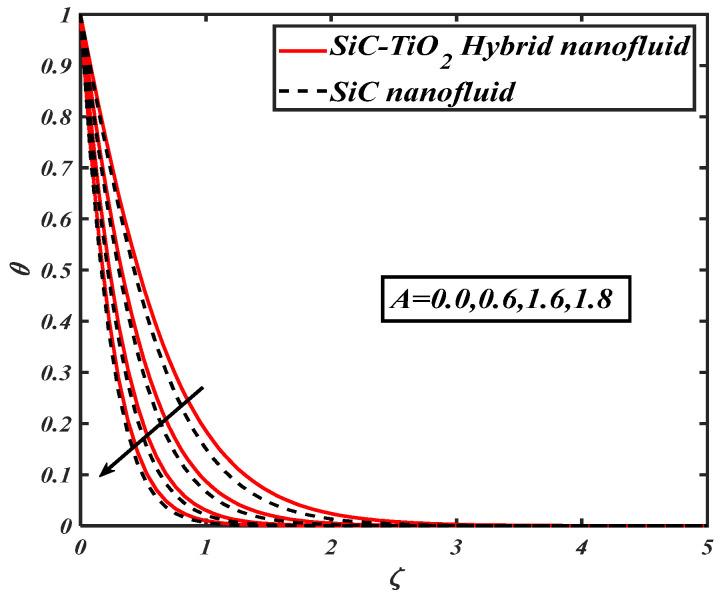
θ for varied A.

**Figure 7:**
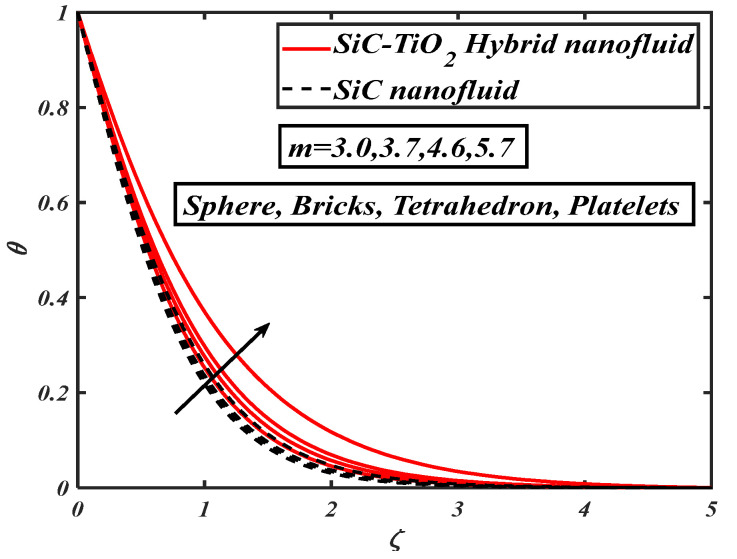
θ for different shapes factors.

**Figure 8:**
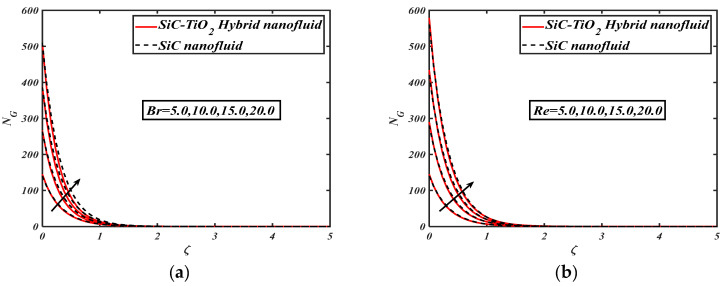
(**a**) $N_{G}$ for varied Br, and (**b**) $N_{G}$ for varied Re.

The quantities significantly changed the behavior of flow through the desired domain. This section marks the parameters controlling characteristics of flow against flow and heat transfer of fluid. In the current article, a numerical solution is obtained by applying the bvp4c tool. Figure 2, Figure 3, Figure 4, Figure 5, Figure 6, Figure 7 and Figure 8 examine the performance of parameters across subjective fields. The current results are compared with Rafiq et al. [31] and Jamshed et al. [32] as described in Table 3. Table 1 contains the thermophysical properties of solid particles and base fluid. The results are computed for an ample range of prominent parameters as 0.1≤S≤1.2,0.0≤A≤1.8, $0.01\leq \phi_{1}=\phi_{2}\leq 0.04,0.1\leq Ec\leq 1.2,$ m=3.0,m=3.7,m=4.6, m=5.7,5.0≤Br≤20.0, 5.0≤Re≤20.0.

Figure 2 analyzes the effect of suction parameters via the velocity of fluid. It is visualized that the velocity profile is reduced for suction parameter values. Figure 3 is considered to show the behavior of the velocity ratio parameter against the fluid velocity profile. The growing estimations of the velocity ratio parameter result in an increment of velocity profile.

Figure 4 illustrates the nature of nanoparticle fraction on a thermal field of species. It is noted that the thermal distribution increases as the fraction of nanoparticles escalates. Figure 5 signifies the heat transfer field for increasing Eckert number. It is observed that larger values of the Eckert number boost the temperature of hybrid nanofluids. Physically, enhancement in the Eckert number improves the thermal state of fluid. Figure 6 examines the effect of velocity ratio parameter on thermal field. It can be noted that the temperature of fluid decreases when the values of the velocity ratio parameter are increased. Figure 7 illustrates the behavior of different shape factors, namely, sphere, bricks, tetrahedron and platelets, versus the temperature profile. It is noted that different variations of shape factors improve the energy profile of the hybrid nanofluid.

Figure 8a allows an inspection of the performance of the Brinkman number via an entropy generation field. Here, we suggest that entropy generation is boosted via the Brinkman number. The Brinkman number describes the viscosity and conductivity ratio among the medium, and heat variance between the surface and the ambient temperature. The entropy of system increases with increases in the medium conductivity via larger Brinkman numbers. Figure 8b indicates the trend in the Reynolds number via entropy generation. The entropy generation is an enhancing function of the Reynolds number. It is evident that the greater Reynolds number is due to a smaller viscous force that promotes the flow irreversibility of the structure.

## 5. Conclusions

Two-dimensional flow of kerosene oil in existence of SiC and $TiO_{2}$ nanoparticles past a moving flat horizontal surface with different shape effects and entropy production is studied numerically in this article. The tool of similarity transformations of variables is utilized to simplify flow governing the system of PDEs, and a bvp4c solver with the shooting technique utilized for solution development. The thermal profile of species improved with growing values of the Eckert number. The temperature profile escalated by enlarging the fraction of nanoparticles. The entropy production can be boosted with the enhancement of the Brinkmann parameter. The increment of Reynolds number augments the entropy production.

## Figures and Tables

**Table 1:** Thermophysical properties of base fluid with nanoparticles.

Physical Characteristics	Kerosene Oil	SiC	$TiO_{2}$
$C_{p}/JKg^{-1}K^{-1}$	2090	1340	686.2
$\rho /Kgm^{-3}$	783	3370	4250
$k/Wm^{-1}K^{-1}$	0.15	150	8.9638
$\sigma /\Omega^{-1}m^{-1}$	$5\times 10^{-11}$	--	$2.38\times 10^{6}$

**Table 2:** Geometrical manifestation of tiny-sized particles with their size and sphericity.

Nanoparticle Shape	Geometrical Appearance	Size	Sphericity
Sphere	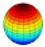	3.0	1.0
Tetrahedron	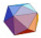	4.0613	0.82
Bricks	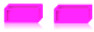	3.7	0.81
Platelets	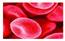	5.7	0.52

**Table 3:** Validation of results in the case of pure fluid.

Γ	S	CfRex12		
		Rafiq et al. [31]	Jamshed et al. [32]	Current Results
0.0	0.5	−6.15999842542	−6.15983	−6.15982
0.2	0.5	−4.81951263596	−4.81947	−4.81945
0.5	0.0	−2.24678804753	−2.24666	−2.24664

## Data Availability

The data that support the findings of this study have not been made available but can be obtained from the author upon request.

## Nomenclature

$\left(u\&v\right)$	Velocity components $\left[m.s^{-1}\right]$
$\left(x\&y\right)$	Cartesian coordinates $\left[m\right]$
$\left(\alpha_{1},\alpha_{2}\right)$	Material constants
$\left(U_{w}\right)$	Stretching velocity $\left[m.s^{-1}\right]$
μ	Dynamic viscosity $\left(kg/ms\right)$
p	Pressure
$\left(A_{\zeta 1}\&A_{\zeta 2}\right)$	Rivlin–Erickson tensors
I	Identity tensor
$\frac{d}{dt}$	Time-dependent derivative
V	Velocity of fluid
$\rho_{hnf}$	Density of hybrid nanofluid $\left(kg/m^{3}\right)$
$k_{hnf}$	Thermal conductivity of hybrid nanofluid $\left(Wm^{-1}K^{-1}\right)$
${\left(\rho C_{p}\right)}_{hnf}$	Hybrid nanofluid heat capacity $\left(Jm^{-3}K^{-1}\right)$
$C_{p}$	Specific heat $\left(J/K\right)$
$q_{r}$	Radiative heat flux $\left(W/m^{2}\right)$
B	Strength of Magnetic field $\left[N.m^{-1}.A^{-1}\right]$
$\sigma^{*}$	Stefan–Boltzmann constant
$k^{*}$	Mean absorption coefficient
Γ	Second grade fluid parameter
K	Porousity parameter
Nr	Thermal radiation parameter
Pr	Prandtl number
Ec	Eckert number
M	Magnetic parameter
S	Suction/injection velocity $\left(ms^{-1}\right)$
A	Velocity ratio parameter
$\phi_{1},\phi_{2}$	Volume fraction of nanoparticles
$T_{w}$	Temperature of the surface $\left(K\right)$
$T_{\infty }$	Neighborhood temperature $\left(K\right)$
$C_{f}$	Coefficient of skin friction
$Nu_{x}$	Nusselt number
$\tau_{w}$	Shear stress
$q_{w}$	Heat flux
Rex	Local Reynolds number
Br	Brinkman number
Ω	Temperature gradient
$N_{G}$	Entropy generation
hnf	Thermophysical properties defined for hybrid nanofluid
